# Estimated Glucose Disposal Rate Associated With Risk of Frailty and Likelihood of Reversion

**DOI:** 10.1002/jcsm.13814

**Published:** 2025-04-17

**Authors:** Dingchun Hou, Shangjun Liu, Yumei Sun, Chang Liu, Xue Shang, Lijun Pei, Gong Chen

**Affiliations:** ^1^ Institute of Population Research Peking University Beijing China; ^2^ School of Nursing Peking University Beijing China; ^3^ School of Sport Science Beijing Sport University Beijing China; ^4^ Institute of Ageing Studies Peking University Beijing China

**Keywords:** estimated glucose disposal rate, frailty, insulin resistance, reversibility

## Abstract

**Background:**

Estimated glucose disposal rate (eGDR) is a simple and effective measure for insulin resistance, which is associated with higher risk of frailty. We aim to analyse the associations of eGDR with frailty risk and its reversibility.

**Methods:**

A population‐based longitudinal study was conducted of 11 670 participants from the China Health and Retirement Longitudinal Study and 19 355 participants from the Health and Retirement Study. Frailty was assessed by the frailty index and reversibility was measured by transitions from frailty at baseline to non‐frailty during follow‐up. The eGDR was divided into Q_1_, Q_2_, Q_3_ and Q_4_ according to the quartiles. Multi‐state Markov model was performed to evaluate the effects of eGDR on transitions among non‐frailty, frailty and death. Cox regression model was used to estimate eGDR associated with the risk of frailty and the likelihood of reversion.

**Results:**

In Chinese population characterized by a median age of 60 years (IQR: 54–66) with 6119 women (52.43%), compared with the Q_1_ level of eGDR, participants exposure to Q_3_ and Q_4_ level decreased the probability of transitioning from non‐frailty to frailty by 22% (HR = 0.78, 95% CI: 0.69–0.88) and 25% (HR = 0.75, 95% CI: 0.66–0.86), respectively. But its Q_2_, Q_3_ and Q_4_ levels increased the probability of transitioning from frailty to non‐frailty by 24% (HR = 1.24, 95% CI: 1.06–1.44), 39% (HR = 1.39, 95% CI: 1.19–1.64) and 33% (HR = 1.33, 95% CI: 1.13–1.58). In American population with a median age of 63 years (IQR: 56–72) and 11 189 women (57.81%), its Q_2_, Q_3_ and Q_4_ levels decreased the probability of transitioning from non‐frailty to frailty by 17% (HR = 0.83, 95% CI: 0.77–0.89), 24% (HR = 0.76, 95% CI: 0.70–0.82) and 46% (HR = 0.54, 95% CI: 0.49–0.59), respectively. The probability of revising frailty increased by 25% (HR = 1.25, 95% CI: 1.13–1.38), 36% (HR = 1.36, 95% CI: 1.22–1.51) and 48% (HR = 1.48, 95% CI: 1.30–1.69) for levels Q_2_, Q_3_ and Q_4_. As shown in the prospective analysis, increased eGDR levels from Q_2_ to Q_4_ were associated with decreased frailty risk and higher likelihood of reversion, as evidenced by the dose–response relationship revealed by restricted cubic spline analysis.

**Conclusions:**

Higher levels of eGDR were associated with a reduced risk of frailty, delayed transition from non‐frailty to frailty and an increased likelihood of reversion. eGDR emerges as a promising predictor for early frailty detection, prognosis assessment and a potential therapeutic target for intervention strategies.

**Abbreviations**
BMI: body mass index
CHARLS: China Health and Retirement Longitudinal Study
CI: confidence interval
CRP: C‐reactive protein
eGDR: estimated glucose disposal rate
FI: frailty index
HbA1c: glycosylated haemoglobin A1c
HDL‐C: high‐density lipoprotein cholesterol
HEGC: hyperinsulinaemic‐euglycaemic clamp
HOMA‐IR: homeostasis model assessment of insulin resistance
HR: hazard ratio
HRS: Health and Retirement Study
RCS: restricted cubic spline
TC: total cholesterol
TyG: triglyceride‐glucose
WC: waist circumference

## 1 Introduction

Frailty, a dynamic degenerative ageing process associated with age, is characterized by a condition of diminished physiological reserve of numerous systems and reduced physical and mental resilience to stress [1, 2]. Even in young adults, frailty is strongly linked to mortality and is a reflection of biological and phenotypic factors rather than chronological age [3, 4]. And frailty risk increases with age, significantly increasing the susceptibility to adverse health outcomes such as falls, disability and mortality [1, 2]. However, in certain patients, especially in the early stages, frailty may be reversible [1, 2, 3]. Therefore, prognosis, clinical care and interventions planning may be affected if frailty and its modifiable associated factors are identified in middle‐aged and older populations.

Insulin resistance (IR) is defined physiologically as a state of reduced sensitivity or responsiveness in insulin‐targeting tissues to insulin and is considered the pathogenic driver of a spectrum of chronic diseases, such as Type 2 diabetes and hypertension [5]. Prospective cohort studies have illuminated the association between IR and an increased frailty risk [6, 7]. The gold standard for measuring IR, hyperinsulinaemic‐euglycaemic clamp (HEGC), is unsuitable for broad clinical application and large‐scale epidemiological studies for its invasiveness, cost and complexity. Then homeostasis model assessment of insulin resistance (HOMA‐IR) was developed for clinical studies; however, it is susceptible to exogenous insulin therapy and is not suitable for patients with β‐cell incompetence [8].

Insulin resistance syndrome is characterized by hyperglycaemia, abdominal obesity, dyslipidaemia and hypertension [9]. Underlying mechanisms emphasize the links between these clinical symptoms and IR, including ectopic lipid accumulation and endothelial dysfunction reflecting nitric oxide deficiency [5, 9]. Thus, estimated glucose disposal rate (eGDR), calculated from clinically accessible parameters including waist circumference, blood pressure status and glycated haemoglobin, offers a novel, practical and simple approach to assessing IR in a non‐invasive manner [10, 11, 12]. A study indicated that eGDR exhibited superior predictive performance for both risk and progression of frailty, compared to other non‐insulin‐based IR indicators [7]. Notably, eGDR has been proven associated with cardiovascular events and mortality in populations with Type 1 or 2 diabetes, and non‐diabetic chronic kidney disease, indicating its potential role in assessing broader health outcomes beyond just IR [11, 12, 13]. Consequently, eGDR could be a valuable marker for further investigation in this context. And although previous studies have concentrated on the associations between IR and the risk or progression of frailty, there is a growing interest in understanding the effect of IR on its reversibility [6, 7, 14]. Although gait speed abnormalities are a significant component of frailty, there remains a scarcity of research addressing the association between IR and directly measured frailty reversibility [14]. The reversibility of frailty is a critical area of research, and eGDR could offer valuable insights into the modifiable factors associated with frailty outcomes. It is significant to focus on elucidating the role of eGDR in the frailty reversibility and its potential as a therapeutic target for interventions aimed at improving health outcomes in frail populations.

Given that frailty and IR have been major challenges for global public health, it is of great referential significance to conduct research in China and the United States, two countries with significant regional and global influence. On the one hand, the health and economic burdens of frailty and metabolic diseases are heavy in both two countries [4, 15]. On the other hand, significant cultural, social and healthcare system differences exist between them, which can provide reference for developing and developed countries, respectively. In order to provide reference for the study of pathophysiological mechanisms, early prevention and clinical management of frailty, this study estimated the associations of eGDR with the risk of frailty and the likelihood of reversion in middle‐aged and older populations using two nationwide population‐based longitudinal survey databases from China and the United States.

## 2 Methods

### 2.1 Study Population

A population‐based longitudinal study was conducted in this study. The data were from the China Health and Retirement Longitudinal Study (CHARLS) and Health and Retirement Study (HRS) from the United States, and detailed information of study design for two longitudinal surveys have been published previously [16, 17]. In brief, two nationwide, prospective, population‐based longitudinal studies, CHARLS and HRS, were approved by the Ethics Review Committees of Peking University and the University of Michigan, respectively. Informed consent was obtained from each participant in these two surveys.

In the CHARLS, Wave 1 (2011) and Wave 3 (2015) were considered the baseline. Subsequent follow‐up surveys were conducted to track outcomes until Wave 4 (2018). In the HRS, Wave 8 (2006) to Wave 13 (2016) were used as the baseline, with follow‐up surveys continuing until Wave 15 (2020). Participants were recruited if they were aged 50 years or older and undergone blood tests and physical examinations at baseline. Individuals with missing data for 10% or more items of frailty index at baseline or during follow‐up were excluded. Finally, 11 670 participants from the CHARLS and 19 355 participants from the HRS were included in the multi‐state transition analysis between eGDR and frailty.

In the CHARLS, after excluding 3307 participants who were frail at baseline, 8363 participants were included for the prospective analysis examining the association between eGDR and frailty risk. The 3307 participants who were frail at baseline were included in the analysis assessing the association between eGDR and the reversibility of frailty. In the HRS, after excluding 4260 participants who were frail at baseline, 15 095 participants were included for the prospective analysis examining the association between eGDR and frailty risk. The 4260 participants who were frail at baseline were included in the analysis assessing the association between eGDR and frailty reversibility. The flowcharts depicting the sample selection process are shown in Figure S1 and S2.

### 2.2 Assessment of Frailty

Frailty was assessed using the frailty index (FI), which is calculated based on the accumulation of age‐related health deficits [6, 18]. In this study, we constructed the FI in accordance with the standard procedures previously described [19]. After screening the data from the CHARLS and HRS, we selected 30 items to construct the FI, encompassing variables related to comorbidity (excluding hypertension), physical function, disability, depression and cognition (Data S1). Each item was dichotomized into 0 or 1 based on specific cut‐off values, with the exception of the cognition item. A value of 0 indicated the absence of a deficit, whereas 1 indicated its presence. The cognition item was treated as a continuous variable, ranging from 0 to 1, where a higher value signified poorer cognitive function. Consistent with previous studies, the FI was used to categorize participants into two groups: non‐frailty (FI < 0.25) and frailty (FI ≥ 0.25) [18]. The FI was calculated for each wave of both the CHARLS and HRS datasets. Given the low proportion of missing data for each FI item, random forest imputation using the missRanger package in R software was employed to maximize the sample size [20]. The reversibility of frailty was assessed by tracking transitions from a state of frailty at baseline to a state of non‐frailty during the follow‐up period.

### 2.3 Access to Death Data

The date of death for participants was ascertained through interviews with family members, who were contacted at each follow‐up investigation.

### 2.4 Assessment of Estimated Glucose Disposal Rate

The estimated glucose disposal rate (eGDR), an exposure factor at baseline, was calculated using the following formula: eGDR (mg/kg/min) = 21.158 − (0.09 × WC) − (3.407 × hypertension) − (0.551 × HbA1c), where WC represents waist circumference in centimetres, hypertension is coded as yes = 1/no = 0 and HbA1c is glycosylated haemoglobin A1c in per cent [10, 12]. A lower eGDR level indicates greater insulin resistance.

### 2.5 Assessment of Covariates

The covariates included age, sex, marital status, educational level, smoking status, alcohol consumption, body mass index (BMI, calculated as weight in kilograms divided by the square of height in meters), hand grip strength, pain, C‐reactive protein (CRP), high‐density lipoprotein cholesterol (HDL‐C), total cholesterol (TC) and diabetic status. To ensure consistency in covariate classification between the CHARLS and HRS, marital status was categorized as married or partnered versus others (unmarried, separated, divorced or widowed). Educational level was divided into two categories: below high school and high school or above. Smoking status was categorized into three groups: current smokers, former smokers and never smokers. Alcohol consumption was dichotomized into yes and no. BMI was classified into three categories: underweight or normal (< 25), overweight (≥ 25 and < 30) and obese (> 30). Diabetic status was determined based on the presence of diabetes and the receipt of diabetes‐related treatment, which included insulin injections, oral medications or other therapeutic interventions. Participants were subsequently stratified into three distinct categories: non‐diabetic, diabetic with treatment and diabetic without treatment. The treatment for diabetes included taking Chinese traditional medicine, Western modern medicine or insulin injections in the CHARLS and taking oral medication or using insulin shots or a pump in the HRS. Data for all covariates were collected at baseline. Given the low proportion of missing data for covariates, random forest imputation using missRanger package in R software was applied [20].

### 2.6 Statistical Analysis

Participants were stratified into four groups based on the quartiles of eGDR at baseline [12]. For descriptive analysis, continuous variables that were not normally distributed were presented as median and interquartile range (IQR). Categorical variables were presented as frequency and percentage.

In this study, we performed statistical inference through three distinct steps. First, three‐state Markov models, which are effective for describing processes where individuals transition between states over continuous time, were utilized for multi‐state analysis. These models included states of non‐frailty, frailty and death and were employed to evaluate the reversibility of frailty using mean sojourn times and transition probabilities at 1‐year, 5‐year and 10‐year intervals [21]. The Markov models were also used to estimate the associations between eGDR and transitions among non‐frailty, frailty and death states.

Second, to mitigate the risk of reverse causality, we used the Cox proportional hazard regression model to estimate the association between eGDR at baseline as an exposure and subsequent frailty as the outcome in the longitudinal data (Analysis 2).

Finally, to confirm the association between eGDR and frailty reversibility, we constructed prospective cohorts with non‐frailty as the outcome and applied the Cox proportional hazard regression model to estimate the association between eGDR and the probability of non‐frailty (Analysis 3).

Furthermore, restricted cubic splines (RCS) with four knots were employed to explore the dose–response relationships between eGDR and the risk and reversibility of frailty. To ensure the robustness of the models, we winsorized eGDR as a continuous variable, applying a two‐sided 1% trim. Given that eGDR may interact with covariates, we conducted subgroup analyses. All models referenced the Q_1_ level of eGDR, adjusted for baseline covariates and presented results as hazard ratios (HR) with 95% confidence intervals (95% CI). For additional robustness checks, especially for the Markov models, we transformed continuous covariates such as hand grip strength, CRP, HDL‐C and TC into categorical variables based on their quartiles.

To ascertain the robustness of our findings, we conducted several sensitivity analyses: (1) Given that eGDR can be influenced by obesity, hyperglycaemia and hypertension, we utilized WC, HbA1c and blood pressure as the exposures to repeat our analyses. (2) We utilized a revised FI that excluded self‐reported diabetes from the original FI to avoid potential overlap with eGDR. (3) We excluded participants who had diabetes at baseline.

All analyses were performed using STATA, Version 17.0, and R software, Version 4.4.1. *p* < 0.05 (two‐tailed) was considered statistically significant.

## 3 Results

### 3.1 Baseline and Follow‐Up Characters of Study Populations

A total of 11 670 eligible Chinese participants with a median age of 60 (IQR: 54–66) and 19 355 eligible American participants with a median age of 63 (IQR: 56–72) were included in this analysis. Among Chinese participants, we observed 3286 transitions from non‐frailty to frailty and 1854 transitions from frailty to non‐frailty across 4 waves; 268 and 324 participants transitioned to death from non‐frailty and frailty, respectively. Among American participants, we observed 6684 transitions from non‐frailty to frailty and 3451 transitions from frailty to non‐frailty across 8 waves; 1512 and 2937 participants transitioned to death from non‐frailty and frailty, respectively. For Analysis 2, 8363 eligible Chinese participants were included, with an average follow‐up of 4.59 person‐years and an incidence density of frailty at 67.11 per 1000 person‐years. For American participants, 15 095 were included, with an average follow‐up of 7.64 person‐years and an incidence density of frailty at 42.88 per 1000 person‐years. For Analysis 3, 3307 eligible Chinese participants were included, with an average follow‐up of 4.08 person‐years and an incidence density of non‐frailty at 93.24 per 1000 person‐years. And 4260 eligible American participants were included, with an average follow‐up of 5.76 person‐years and an incidence density of non‐frailty at 54.86 per 1000 person‐years. The baseline characters of the study populations for the multi‐state analysis are detailed in Table 1.

**TABLE 1:** Distribution characteristics of estimated glucose disposal rate among Chinese and American population at baseline.

Outcomes/covariates	Chinese participants (*n* = 11 670)				American participants (*n* = 19 355)			
	Estimated glucose disposal rate				Estimated glucose disposal rate			
	Q_1_	Q_2_	Q_3_	Q_4_	Q_1_	Q_2_	Q_3_	Q_4_
	*n* (%)	*n* (%)	*n* (%)	*n* (%)	*n* (%)	*n* (%)	*n* (%)	*n* (%)
Sample	2917	2917	2918	2918	4836	4837	4833	4849
Estimated glucose disposal rate (mg/kg/min), median (IQR)	6.29 (5.63, 6.86)	9.26 (8.37, 9.70)	10.55 (10.31, 10.79)	11.50 (11.25, 11.83)	3.87 (2.97, 4.46)	5.78 (5.36, 6.21)	8.08 (7.40, 8.69)	10.13 (9.64, 10.78)
Age								
≥ 50 and < 65 years	1877 (64.35)	2048 (70.21)	2089 (71.59)	1996 (68.40)	2532 (52.36)	2186 (45.19)	2587 (53.52)	3127 (64.48)
≥ 65 and < 75 years	787 (26.98)	660 (22.63)	633 (21.69)	673 (23.06)	1403 (29.01)	1422 (29.40)	1254 (25.95)	1038 (21.41)
≥ 75 years	253 (8.67)	209 (7.16)	196 (6.72)	249 (8.54)	901 (18.63)	1229 (25.41)	992 (20.53)	684 (14.11)
Sex								
Male	1267 (43.44)	1321 (45.29)	1388 (47.57)	1575 (53.98)	2324 (48.06)	2078 (42.96)	2177 (45.04)	1587 (32.73)
Female	1650 (56.56)	1596 (54.71)	1530 (52.43)	1343 (46.02)	2512 (51.94)	2759 (57.04)	2656 (54.96)	3262 (67.27)
Marital status								
Married or partnered	2528 (86.66)	2553 (87.52)	2570 (88.07)	2527 (86.60)	3112 (64.35)	3183 (65.81)	3307 (68.43)	3420 (70.53)
Others	389 (13.34)	364 (12.48)	348 (11.93)	391 (13.40)	1724 (35.65)	1654 (34.19)	1526 (31.57)	1429 (29.47)
Educational level								
Below high school	2596 (89.00)	2579 (88.41)	2610 (89.44)	2672 (91.57)	1413 (29.22)	1265 (26.15)	1135 (23.48)	861 (17.76)
High school and above	321 (11.00)	338 (11.59)	308 (10.56)	246 (8.43)	3423 (70.78)	3572 (73.85)	3698 (76.52)	3988 (82.24)
Smoking status								
Never	1830 (62.74)	1757 (60.23)	1715 (58.77)	1505 (51.58)	1969 (40.72)	2156 (44.57)	2080 (43.04)	2262 (46.65)
Former	423 (14.50)	355 (12.17)	317 (10.87)	241 (8.26)	2212 (45.74)	1962 (40.56)	1910 (39.52)	1684 (34.73)
Current	664 (22.76)	805 (27.60)	886 (30.36)	1172 (40.16)	655 (13.54)	719 (14.87)	843 (17.44)	903 (18.62)
Alcohol consumption								
No	2080 (71.31)	1937 (66.40)	1887 (64.67)	1824 (62.51)	2448 (50.62)	2188 (45.23)	1976 (40.89)	1704 (35.14)
Yes	837 (28.69)	980 (33.60)	1031 (35.33)	1094 (37.49)	2388 (49.38)	2649 (54.77)	2857 (59.11)	3145 (64.86)
BMI								
Underweight or normal	1174 (40.24)	1396 (47.86)	2367 (81.12)	2860 (98.01)	53 (1.10)	651 (13.46)	991 (20.50)	2281 (47.04)
Overweight	1361 (46.66)	1297 (44.46)	528 (18.09)	49 (1.68)	626 (12.94)	2309 (47.73)	1657 (34.29)	2071 (42.71)
Obese	382 (13.10)	224 (7.68)	23 (0.79)	9 (0.31)	4157 (85.96)	1877 (38.81)	2185 (45.21)	497 (10.25)
Pain								
No	1869 (64.07)	1963 (67.30)	1954 (66.96)	1938 (66.42)	2563 (53.00)	3034 (62.72)	3262 (67.49)	3559 (73.40)
Yes	1048 (35.93)	954 (32.70)	964 (33.04)	980 (33.58)	2273 (47.00)	1803 (37.28)	1571 (32.51)	1290 (26.60)
Hand grip strength (kg), median (IQR)	30.00 (24.10, 38.50)	31.00 (24.50, 39.00)	31.00 (25.00, 39.00)	30.50 (24.60, 38.00)	31.50 (25.00, 41.39)	30.00 (23.95, 39.50)	31.00 (25.00, 42.00)	30.00 (24.50, 38.00)
CRP (mg/L), median (IQR)	1.70 (0.90, 3.20)	1.36 (0.72, 2.60)	1.06 (0.60, 2.08)	0.80 (0.47, 1.79)	3.37 (1.56, 7.24)	2.17 (1.04, 4.73)	2.06 (0.96, 4.38)	1.28 (0.58, 2.93)
HDL‐C (mg/dL), median (IQR)	45.95 (39.05, 53.74)	47.88 (40.54, 56.83)	50.97 (42.53, 59.15)	55.28 (46.39, 64.86)	49.14 (40.96, 57.98)	52.43 (44.03, 62.78)	53.28 (44.32, 63.36)	58.90 (49.01, 70.44)
TC (mg/dL), median (IQR)	192.28 (169.11, 218.43)	190.35 (168.17, 214.56)	188.42 (165.25, 212.63)	181.70 (159.85, 205.67)	190.00 (164.73, 218.15)	195.23 (167.78, 224.87)	200.28 (173.52, 227.61)	206.67 (179.62, 234.77)
Diabetes status								
Non‐diabetic	2392 (82.00)	2662 (91.25)	2850 (97.67)	2877 (98.59)	2629 (54.36)	3940 (81.46)	4259 (88.12)	4693 (96.78)
Diabetic with treatment	381 (13.06)	179 (6.14)	31 (1.06)	18 (0.62)	1980 (40.94)	742 (15.34)	489 (10.12)	113 (2.33)
Diabetic without treatment	144 (4.94)	76 (2.61)	37 (1.27)	23 (0.79)	227 (4.70)	155 (3.20)	85 (1.76)	43 (0.89)
Waist circumference (cm), median (IQR)	93.00 (87.70, 99.20)	92.50 (83.40, 97.20)	85.00 (82.20, 88.00)	75.20 (72.00, 78.30)	115.57 (109.86, 124.46)	99.06 (93.98, 104.14)	102.87 (89.54, 109.22)	88.90 (82.55, 93.98)
HbA1c (%), median (IQR)	5.60 (5.20, 6.10)	5.50 (5.20, 6.00)	5.40 (5.00, 5.70)	5.10 (4.80, 5.40)	6.15 (5.60, 6.98)	5.69 (5.34, 6.06)	5.60 (5.34, 6.02)	5.36 (5.11, 5.69)
Hypertension								
No	104 (3.57)	2210 (75.76)	2918 (100.00)	2918 (100.00)	39 (1.48)	251 (4.78)	1381 (34.05)	7263 (97.92)
Yes	2813 (96.43)	707 (24.24)	0 (0.00)	0 (0.00)	2591 (98.52)	5001 (95.22)	2675 (65.95)	154 (2.08)
Systolic blood pressure (mmHg), median (IQR)	140.50 (127.50, 153.00)	128.50 (116.50, 142.00)	121.50 (111.00, 134.00)	118.50 (108.00, 131.50)	132.50 (120.50, 145.50)	131.50 (119.50, 145.00)	127.00 (116.50, 140.50)	120.50 (109.50, 133.50)
Diastolic blood pressure (mmHg), median (IQR)	81.00 (73.00, 89.00)	75.50 (68.50, 83.50)	72.00 (65.50, 79.50)	70.00 (63.50, 77.50)	81.50 (74.00, 89.00)	80.50 (73.00, 88.00)	80.00 (72.50, 87.50)	77.00 (70.00, 84.00)
FI, median (IQR)	0.21 (0.12, 0.31)	0.17 (0.11, 0.28)	0.15 (0.09, 0.25)	0.15 (0.09, 0.24)	0.19 (0.11, 0.32)	0.14 (0.08, 0.25)	0.11 (0.05, 0.21)	0.08 (0.04, 0.15)
Non‐frailty	1846 (63.28)	2050 (70.28)	2224 (76.22)	2243 (76.87)	3046 (62.99)	3656 (75.58)	4028 (83.34)	4365 (90.02)
Frailty	1071 (36.72)	867 (29.72)	694 (23.78)	675 (23.13)	1790 (37.01)	1181 (24.42)	805 (16.66)	484 (9.98)

Abbreviations: BMI, body mass index; CRP, C‐reactive protein; eGDR, estimated glucose disposal rate; FI, frailty index; HbA1c, glycosylated haemoglobin A1c; HDL‐C, high‐density lipoprotein cholesterol; TC, total cholesterol.

### 3.2 Associations of eGDR and the Progression and Reversibility of Frailty

According to the Markov models, the mean sojourn time in each state before any transition occurred was 7.99 (95% CI: 7.68–8.31) years for non‐frailty and 5.43 (95% CI: 5.14–5.73) years for frailty among Chinese participants. For American participants, these times were 12.17 (95% CI: 11.87–12.47) years and 5.37 (95% CI: 5.19–5.55) years, respectively. Among Chinese participants, the probability of remaining non‐frailty decreased from 89.15% to 57.90% over 10 years, whereas the probability of remaining frailty decreased from 84.06% to 40.19%. The probability of transitioning from non‐frailty to frailty increased from 10.53% to 36.64%, and the probability of transitioning from non‐frailty to death increased from 0.32% to 5.46%. Similarly, the probability of transitioning from frailty to death increased from 1.24% to 8.67%.

Among American participants, the probability of remaining non‐frailty decreased from 92.56% to 61.37% over 10 years, and the probability of remaining frailty decreased from 83.43% to 27.98%. The probability of transitioning from non‐frailty to frailty increased from 6.82% to 24.94%, and the probability of transitioning from non‐frailty to death increased from 0.62% to 13.69%. The probability of transitioning from frailty to death increased from 5.22% to 30.50%. Notably, the probability of transitioning from frailty to non‐frailty increased for both Chinese and American participants (Table 2).

**TABLE 2:** 1‐year, 5‐year and 10‐year transition probabilities among non‐frailty, frailty and death among Chinese and American population.

Transitions	Chinese participants (*n* = 11 670)			American participants (*n* = 19 355)		
	Transition probability			Transition probability		
	1‐year	5‐year	10‐year	1‐year	5‐year	10‐year
	% (95% CI)	% (95% CI)	% (95% CI)	% (95% CI)	% (95% CI)	% (95% CI)
State 1 to State 1	89.15 (88.76–89.52)	66.77 (65.84–67.66)	57.90 (56.71–59.01)	92.56 (92.38–92.73)	73.53 (72.98–74.06)	61.37 (60.62–62.11)
State 1 to State 2	10.53 (10.16–10.92)	30.89 (30.02–31.79)	36.64 (35.55–37.70)	6.82 (6.65–6.99)	20.95 (20.49–21.43)	24.94 (24.34–25.52)
State 1 to State 3	0.32 (0.25–0.44)	2.34 (2.01–2.80)	5.46 (4.79–6.34)	0.62 (0.56–0.70)	5.52 (5.23–5.84)	13.69 (13.09–14.36)
State 2 to State 1	14.70 (13.96–15.46)	43.12 (41.57–44.59)	51.14 (49.64–52.62)	11.35 (10.95–11.77)	34.90 (33.88–35.88)	41.53 (40.42–42.64)
State 2 to State 2	84.06 (83.26–84.80)	51.84 (50.28–53.38)	40.19 (38.78–41.59)	83.43 (82.93–83.90)	45.46 (44.37–46.52)	27.98 (27.03–28.95)
State 2 to State 3	1.24 (1.04–1.49)	5.05 (4.29–5.96)	8.67 (7.50–10.12)	5.22 (4.93–5.52)	19.64 (18.66–20.66)	30.50 (29.13–31.91)

*Note:* State 1: non‐frailty; State 2: frailty; State 3: death.

Abbreviation: CI, confidence interval.

Then, associations between different levels of eGDR and transitions among non‐frailty, frailty and death were estimated using Markov models (Table 3). Among Chinese participants, compared with the Q_1_ level of eGDR, its Q_3_ and Q_4_ levels decreased the probability of transitioning from non‐frailty to frailty by 22% (HR = 0.78, 95% CI: 0.69–0.88), and 25% (HR = 0.75, 95% CI: 0.66–0.86), respectively. But its Q_2_, Q_3_, and Q_4_ levels increased the probability of transitioning from frailty to non‐frailty by 24% (HR = 1.24, 95% CI: 1.06–1.44), 39% (HR = 1.39, 95% CI: 1.19–1.64) and 33% (HR = 1.33, 95% CI: 1.13–1.58), respectively. However, the associations between eGDR and transitioning from non‐frailty or frailty to death were not statistically significant (*p* > 0.05).

**TABLE 3:** Hazard ratio of estimated glucose disposal rate with transitions among non‐frailty, frailty and death in the three‐state Markov models, HR (95% CI).

Transitions	Chinese participants (*n* = 11 670)				American participants (*n* = 19 355)			
	Estimated glucose disposal rate				Estimated glucose disposal rate			
	Q_1_	Q_2_	Q_3_	Q_4_	Q_1_	Q_2_	Q_3_	Q_4_
State 1 to State 2	Ref.	0.95 (0.85–1.07)	**0.78 (0.69**–**0.88)**	**0.75 (0.66**–**0.86)**	Ref.	**0.83 (0.77**–**0.89)**	**0.76 (0.70**–**0.82)**	**0.54 (0.49**–**0.59)**
State 1 to State 3	Ref.	0.84 (0.36–1.95)	0.93 (0.41–2.12)	1.60 (0.75–3.43)	Ref.	0.93 (0.65–1.32)	0.76 (0.52–1.10)	**0.58 (0.38**–**0.91)**
State 2 to State 1	Ref.	**1.24 (1.06**–**1.44)**	**1.39 (1.19**–**1.64)**	**1.33 (1.13**–**1.58)**	Ref.	**1.25 (1.13**–**1.38)**	**1.36 (1.22**–**1.51)**	**1.48 (1.30**–**1.69)**
State 2 to State 3	Ref.	0.98 (0.71–1.34)	0.91 (0.63–1.33)	1.03 (0.71–1.49)	Ref.	0.92 (0.83–1.02)	1.02 (0.91–1.14)	0.98 (0.84–1.13)

*Note:* Boldface: *p* < 0.05; State 1: non‐frailty; State 2: frailty; State 3: death.

Abbreviations: CI, confidence interval; eGDR, estimated glucose disposal rate; HR, hazard ratio after adjusting for age, sex, marital status, educational level, smoking status, alcohol consumption, body mass index, hand grip strength, pain, C‐reactive protein, high‐density lipoprotein cholesterol, total cholesterol and diabetes status.

Among American participants, compared with the Q_1_ level of eGDR, its Q_2_, Q_3_ and Q_4_ levels decreased the probability of transitioning from non‐frailty to frailty by 17% (HR = 0.83, 95% CI: 0.77–0.89), 24% (HR = 0.76, 95% CI: 0.70–0.82) and 46% (HR = 0.54, 95% CI: 0.49–0.59), respectively. But its Q_2_, Q_3_ and Q_4_ levels increased the probability of transitioning from frailty to non‐frailty by 25% (HR = 1.25, 95% CI: 1.13–1.38), 36% (HR = 1.36, 95% CI: 1.22–1.51), and 48% (HR = 1.48, 95% CI: 1.30–1.69), respectively. And its Q_4_ level decreased the probability of transitioning from non‐frailty to death by 42% (HR = 0.58, 95% CI: 0.38–0.91). But the associations between eGDR and transitioning from frailty to death were not statistically significant (*p* > 0.05).

To further examine the prospective associations between eGDR and the risk and reversibility of frailty, we constructed prospective cohorts with frailty and non‐frailty as outcomes (Table 4). The Cox proportional hazard regression model was employed for these analyses. Among Chinese participants, with the Q_1_ level of eGDR as the reference, its Q_2_, Q_3_ and Q_4_ levels decreased frailty risk by 15% (HR = 0.85, 95% CI: 0.76–0.94), 25% (HR = 0.75, 95% CI: 0.67–0.85) and 31% (HR = 0.69, 95% CI: 0.61–0.78), respectively. Additionally, its Q_2_, Q_3_ and Q_4_ levels increased the probability of non‐frailty by 23% (HR = 1.23, 95% CI: 1.03–1.48), 45% (HR = 1.45, 95% CI: 1.20–1.74) and 57% (HR = 1.57, 95% CI: 1.29–1.92), respectively. Among American participants, its Q_2_, Q_3_ and Q_4_ levels decreased frailty risk by 20% (HR = 0.80, 95% CI: 0.74–0.87), 27% (HR = 0.73, 95% CI: 0.67–0.79) and 52% (HR = 0.48, 95% CI: 0.43–0.54), respectively. Furthermore, its Q_2_, Q_3_ and Q_4_ levels increased the probability of non‐frailty by 25% (HR = 1.25, 95% CI: 1.05–1.48), 52% (HR = 1.52, 95% CI: 1.26–1.82) and 49% (HR = 1.49, 95% CI: 1.23–1.81), respectively.

**TABLE 4:** Associations of estimated glucose disposal rate with frailty risk and its reversibility in the Cox proportional hazard regression models, HR (95% CI).

Estimated glucose disposal rate	Chinese participants		American participants	
	Frailty	Frailty reversibility	Frailty	Frailty reversibility
	(*n* = 8363)	(*n* = 3307)	(*n* = 15 095)	(*n* = 4260)
Q_1_	Ref.	Ref.	Ref.	Ref.
Q_2_	**0.85 (0.76**–**0.94)**	**1.23 (1.03**–**1.48)**	**0.80 (0.74**–**0.87)**	**1.25 (1.05**–**1.48)**
Q_3_	**0.75 (0.67**–**0.85)**	**1.45 (1.20**–**1.74)**	**0.73 (0.67**–**0.79)**	**1.52 (1.26**–**1.82)**
Q_4_	**0.69 (0.61**–**0.78)**	**1.57 (1.29**–**1.92)**	**0.48 (0.43**–**0.54)**	**1.49 (1.23**–**1.81)**

*Note:* Boldface: *p* < 0.05.

Abbreviations*:* CI, confidence interval; HR, hazard ratio after adjusting for age, sex, marital status, educational level, smoking status, alcohol consumption, body mass index, hand grip strength, pain, C‐reactive protein, high‐density lipoprotein cholesterol, total cholesterol and diabetes status.

**FIGURE 1:**
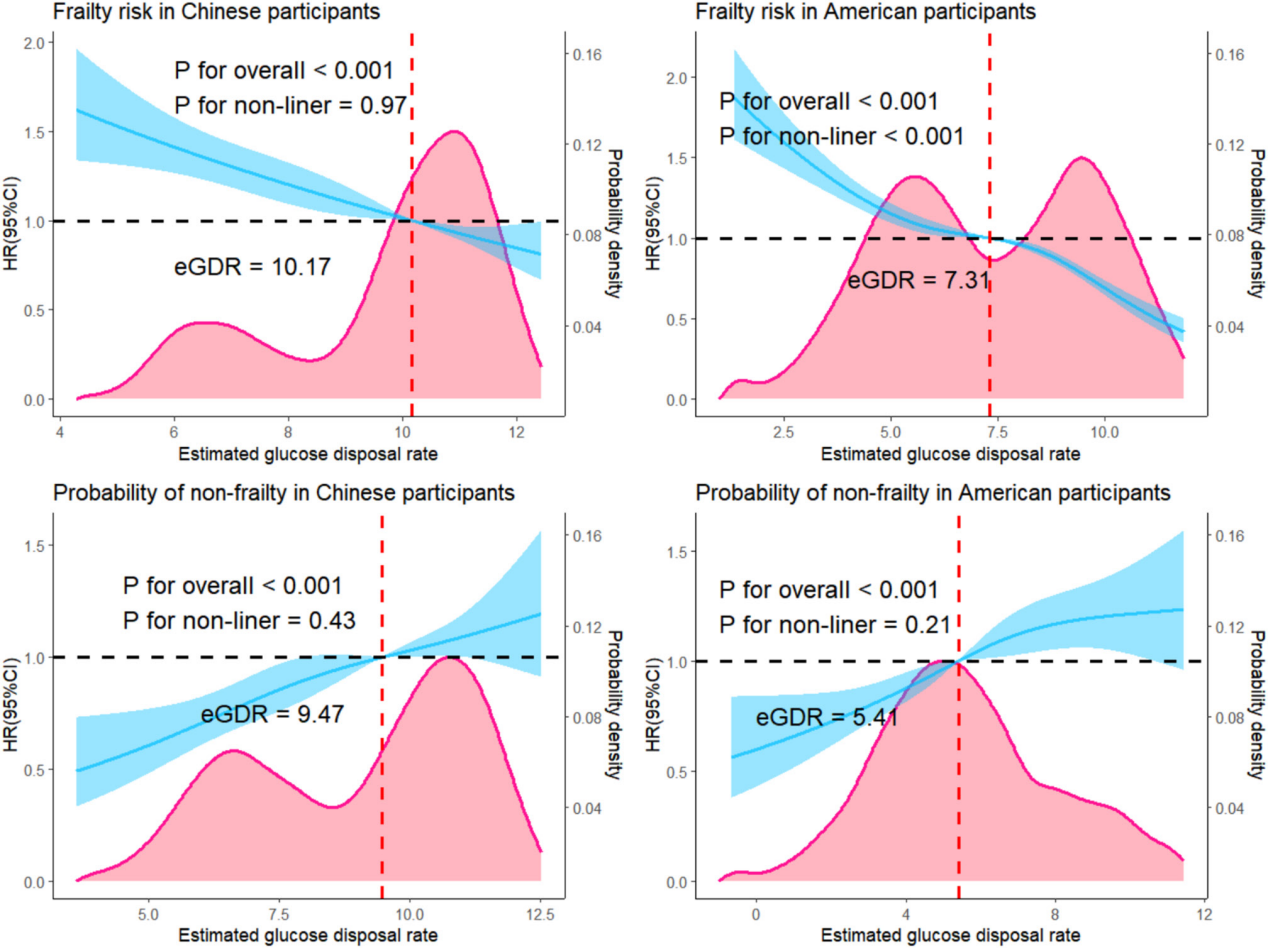
The associations between estimated glucose disposal rate and frailty or non‐frailty in the Chinese and American participants. CI, confidence interval; eGDR, estimated glucose disposal rate; HR, hazard ratio after adjusting for age, sex, marital status, educational level, smoking status, alcohol consumption, body mass index, hand grip strength, pain, C‐reactive protein, high‐density lipoprotein cholesterol, total cholesterol and diabetes status.

Multivariable RCS curves showed dose–response relationships between eGDR as a continuous variable and frailty risk or the probability of non‐frailty (Figure 1). In Chinese participants, the RCS model suggested a negative linear association between eGDR and frailty risk (*p* = 0.97). Conversely, a significant nonlinear association was observed among American participants (*p* < 0.001). The optimal bivariate cut‐off value for frailty risk, at which the HR was equal to 1, was 10.17 mg/kg/min for Chinese and 7.31 mg/kg/min for American participants. Furthermore, the RCS models revealed a positive linear association between eGDR and the probability of non‐frailty in both Chinese (*p* = 0.43) and American (*p* = 0.21) participants. The optimal bivariate cut‐off value for the probability of non‐frailty, with an HR of 1, was 9.47 mg/kg/min for Chinese and 5.41 mg/kg/min for American participants.

According to the subgroup analysis (Figures S3 and S4), in Chinese participants, only pain interacted with eGDR on the frailty risk (*p* = 0.043), and only CRP interacted with eGDR on the frailty reversion (*p* = 0.029). In American participants, age (*p* < 0.001), marital status (*p* = 0.003), BMI (*p* = 0.006), pain (*p* = 0.008), handgrip strength (*p* = 0.029), TC (*p* = 0.001) and diabetic status (*p* = 0.045) interacted with eGDR on the frailty risk, and no significant interaction on the frailty reversion was observed (*p* > 0.05).

### 3.3 Sensitivity Analyses

When we used WC, HbA1c and systolic and diastolic blood pressure as the exposures, there were differences in their associations with frailty between Chinese and American populations (Tables S2 and S3). When we constructed a new FI by excluding self‐reported diabetes or when the analysis was restricted to participants without diabetes at baseline, the results of multi‐state Markov models and prospective analyses were in line with the main analyses (Tables S4–S7). Overall, the sensitivity analyses substantiated the robustness of the main analysis results.

## 4 Discussion

To our best knowledge, this is a large‐scale longitudinal study for association between eGDR and the risk of frailty and the likelihood of reversion, as well as transitions among non‐frailty, frailty and death. Our findings showed that a higher level of eGDR at baseline was associated with a reduced risk of frailty and an increased likelihood of transitioning back from frailty to non‐frailty. Furthermore, a dose–response relationship was observed between eGDR and these outcomes.

Frailty has been observed in populations with insulin resistance [6]. Previous studies have documented the reversibility of frailty [22, 23]. Our study further supports this through multi‐state transition analysis. However, our findings also showed that the probability for transitioning from frailty to non‐frailty increased over a 10‐year period, suggesting that the potential for frailty reversibility may increase with age. This is despite our observation that older age was associated with a higher risk of frailty, and the probability of remaining frailty decreased over time. Similarly, a 12‐year population‐based study on sarcopenia trajectories in older adults showed increased probabilities of transitioning from sarcopenia to non‐sarcopenia and probable sarcopenia over 10 or 5 years [24]. Sarcopenia, an important phenotype of frailty, shares several physiopathologic mechanisms with frailty [22, 25]. Therefore, frailty may represent an unstable pathophysiological state, which provides a theoretical basis for frailty intervention strategies.

Several prospective cohort studies have indicated an association between insulin resistance and an increased risk of frailty [6, 7]. Insulin resistance is likely to play a significant role in the occurrence, development and outcomes of frailty. On the one hand, systemic insulin resistance leads to atherogenic lipid phenotypes by increasing very low‐density lipoprotein particles, which are metabolized into residual lipoproteins that promote atherosclerosis [9, 26, 27]. On the other hand, in the context of hepatic insulin resistance, a typical pathway‐selective insulin responsiveness, insulin fails to suppress hepatic glucose production but stimulates lipogenesis, resulting in hyperglycaemia, hyperlipidaemia and hepatic steatosis [28]. The pro‐inflammatory and procoagulant states induced by insulin resistance also play a crucial role in atherosclerosis formation [9]. Long‐term hyperinsulinemia, hyperglycaemia and hyperlipidaemia can lead to chronic inflammation and oxidative stress [5, 6, 27]. Therefore, IR is a key pathophysiological mechanism in many chronic diseases, including diabetes, hypertension and cardiovascular and cerebrovascular diseases [5, 6]. Meanwhile, the presence of comorbidity is a fundamental phenotype of frailty, accelerating the deterioration of stress resistance. Furthermore, as insulin‐stimulated glucose consumption primarily occurs in skeletal muscle, muscular insulin resistance can impact whole‐body metabolism [5]. In conditions of chronic inflammation and the resultant IR, muscle atrophy is exacerbated, hastening the development of sarcopenia [29]. Given the extensive similarities in clinical outcomes, associations and suggested pathophysiology, sarcopenia is considered a component of frailty, with several metabolic, inflammatory and haematologic markers shared between the two conditions [22, 25]. The interplay between IR and reduced muscle mass creates a detrimental cycle, compromising the regulation of metabolism and physical performance and thereby increasing the susceptibility to frailty.

Obesity and IR are pivotal pathogenic components of the metabolic syndrome, posing a high risk of occurrence in both middle‐aged and older populations [30, 31]. Concurrently, there is a close link between obesity and the development of PI3‐K dysfunctional IR [32]. A noteworthy point is that IR can be reversed, particularly in younger adults [32, 33, 34]. A 9‐year follow‐up study showed that higher levels of insulin were associated with transitions from abnormal to normal gait speed in older adults [14]. This implies a potential role for glucose metabolism in the physiological pathways that influence frailty reversibility, which was supported by our findings. Sarcopenia, a key factor in frailty, also exhibits reversibility. Metabolic disturbances in skeletal muscle cells due to IR, which reduce muscle mass and strength, can be mitigated and potentially reversed through intervention strategies that optimize lifestyle factors [32]. Consequently, frailty reversibility may be significantly enhanced. A 30‐year prospective cohort study demonstrated that long‐term exposure to IR significantly increased frailty risk among young and middle‐aged adults; however, its association with frailty progression—encompassing the transition from non‐frailty to pre‐frailty or frailty, the progression from pre‐frailty to frailty and an increase in the FI among those with frailty at baseline—was not statistically significant [6]. Similarly, our results suggest that eGDR is associated with frailty progression and reversibility. Therefore, maintaining an appropriate level of eGDR could significantly reduce frailty risk and enhance its reversibility in middle‐aged and older populations. eGDR may be suitable for early screening of frailty in large‐scale community populations.

Limited by the invasiveness, cost and complexity of HEGC, other emerging IR metrics have been proposed. HOMA‐IR and triglyceride‐glucose (TyG) index have received wide attention. There are significant differences between eGDR and these two indicators in terms of accuracy, ease of use and applicability to different populations. Strongly associated with the HEGC, eGDR demonstrates high accuracy and its parameters are routine clinical measurements and convenient for application, suitable for both clinical practice and large cohort studies [10, 12, 13]. Meanwhile, eGDR is suitable for the general population and people with multiple diseases and is widely applicable to people of different ages and sexes [8, 10, 12]. But its accuracy may be compromised in populations with complex metabolic disorders. HOMA‐IR is a classic, widely validated and used IR indicator; however, it is difficult to reflect the postprandial and dynamic changes of IR and is susceptible to the influence of exogenous insulin [8]. As for TyG index, it is strongly correlated with other IR indicators, is easy to operate and has good applicability in populations of different races and ages, especially in those with high risk of metabolic syndrome and obesity [7, 35]. However, its accuracy can be affected by hypertriglyceridemia and glucose metabolic disorders [35]. Due to the lack of a standardized definition of IR and heterogeneity among studies, further validation and standardized cut‐off values are required for clinical application [36].

Studies have shown that eGDR may be superior to other IR indicators in predicting frailty and various diseases [7, 8, 11, 13]. A study demonstrated a stronger association of eGDR with frailty progression than TyG index and metabolic score for insulin resistance [7]. Our study further showed that maintaining an appropriate level of eGDR could reduce the risk of frailty and reverse frailty progression. Moreover, eGDR has been proven to be an independent risk factor for first‐time stroke and a significant indicator of stoke outcomes and all‐cause mortality [8, 11]. Compared with its parameters separately, eGDR was more strongly associated with frailty, especially frailty reversion, emphasizing the superiority and necessity of eGDR in evaluating the prognosis and outcome of frailty. Greater attention should be paid to the control of eGDR levels, which is crucial for promoting early prevention of frailty.

Our study revealed a strong consistency in the association between eGDR and both the risk of frailty and the likelihood of reversion between Chinese and American populations, suggesting that their association may have good generalization. The significant dose–response relationships further suggested the robustness and consistency of the associations. China and the United States represent different cultural, socio‐economic and genetic predispositions, profoundly influencing other countries and regions. East Asia is deeply influenced by Confucian culture and shows consistency in social networks, dietary structure and preferences. China can also be used as a social, economic and health reference model for low‐ and middle‐income countries in the rapid development stage. The United States reflects the developed world's health woes. However, population ageing and the burden of frailty and diabetes in these two countries are serious [4]. Studies in them can provide significant reference for solving global public health issues.

There are significant differences in lifestyle, disease spectrum and healthcare system between the two countries. Because the epidemiological transition started more recently in China than in the United States and is happening in a shorter period, the healthcare system in China is not focused on chronic diseases, and their management is far from complete [37]. For example, compared with the United States, China had a lower prevalence of hypertension but a higher mean blood pressure, and China had substantially lower rates of hypertension treatment and control [37]. High‐salt diet and differences in dietary structure may make hypertension an important predisposing factor for IR in China, whereas high‐sugar, high‐calorie diets and sedentary behaviour directly contribute to the health burden of IR in the United States [38]. Our findings showed race differences in the associations of eGDR parameters with frailty. Hypertension may be an important predisposing factor for IR in Chinese population, whereas obesity, especially abdominal obesity, may play a more important role than hypertension in American population. Our subgroup analyses further indicated that pain, associated with several frailty biomarkers, may play an important role in the underlying mechanisms between IR and frailty risk, suggesting that more attention should be paid to the clinical management of pain for the early prevention of frailty [39]. Inflammation reflected by pain and CRP may be a key mechanism affecting insulin resistance and frailty risk or reversion in China, although metabolic diseases represented by obesity may become a priority in the United States [15, 25]. Thus, there may be significant differences in mechanisms between IR and frailty across populations.

Although no significant interaction between eGDR and sex was observed, our study indicated the sex differences in eGDR and its parameters (Table S1). Evidence showed that females typically demonstrated higher insulin sensitivity [14, 40]. And studies have indicated that postmenopausal females are at increased risk for IR and frailty [1, 2, 40]. Although lacking direct evidence supporting the interaction between sex and IR on frailty, it is important to acknowledge that the smaller sample size in the male group of this study may have introduced potential biases in our estimation of the association. In summary, this study carries significant clinical and public health implications. First, our findings affirm the reversibility of frailty, with a decreased risk and increased likelihood of reversion observed in individuals with higher eGDR levels. Additionally, eGDR could serve as a simple, accessible and cost‐effective tool in routine clinical frailty management, especially for early screening and prevention of frailty in resource‐limited settings, providing reference for improving global health. Individuals with low eGDR levels should be prioritized for frailty prevention efforts. Those who are robust or pre‐frail may also benefit from eGDR evaluation to identify at‐risk individuals early, enabling the implementation of timely prevention measures to delay the frailty progression. Furthermore, evaluating eGDR in the frail population, particularly among females, may facilitate the timely determination of frailty prognosis and offer a basis for the development and refinement of intervention programmes.

This study had several strengths. First, the high consistency in findings between the multi‐state transition analysis and the association study across both Chinese and American participants enhanced the reliability of our results. Second, the inclusion of participants from two independent national cohorts with large sample sizes bolstered the generalizability of our findings. Third, by using eGDR as opposed to direct insulin measurements, this study explored the association between eGDR and the risk of frailty and further analysed its association with frailty progression and the likelihood of reversion. This provided a reference for the development of frailty management strategies.

This study also had several limitations that warrant discussion. First, despite the similar design of the two cohorts in both the Chinese and American populations, there were inherent heterogeneities between them. Nonetheless, the highly consistent results across both populations suggested that our findings might be generalized. Second, this study relied on a single baseline assessment of eGDR, which might not adequately capture long‐term insulin resistance and its effect on frailty progression. This highlights the need for future studies to observe the developmental trajectory of eGDR over longer periods. Third, selection bias was present due to sample selection and attrition over the follow‐up period. Finally, there may be unmeasured confounding factors that were not adjusted for in our analyses, potentially introducing confounding bias. For instance, although studies have shown an association between the gene SMP30 and ageing and frailty [39], this gene was not included in our analyses due to the lack of data.

## 5 Conclusions

In conclusion, insulin resistance, as assessed by eGDR, is associated with the risk of frailty, frailty progression and the potential for reversibility in middle‐aged and older populations. Higher levels of eGDR are linked to a reduced risk of frailty, delayed transition from non‐frailty to frailty and an increased likelihood of reversion from frailty. Consequently, eGDR stands out as a promising predictor for early detection of frailty, assessment of prognosis and a potential therapeutic target for the development of intervention strategies.

## Ethics Statement

All human and animal studies have been approved by the appropriate ethics committee and have therefore been performed in accordance with the ethical standards laid down in the 1964 Declaration of Helsinki and its later amendments.

## Consent

Informed consent for publication was obtained from all participants.

## Conflicts of Interest

The authors declare no conflicts of interest.

## Supporting information


**Data S1** Supporting Information.

**Figure S1** Selection flow of the study population in the CHARLS.
**Figure S2** Selection flow of the study population in the HRS.
**Figure S3** Subgroup analysis of the association between estimated glucose disposal rate and the risk of frailty in Chinese and American populations.
**Figure S4** Subgroup analysis of the association between estimated glucose disposal rate and the likelihood of frailty reversion in Chinese and American populations.

**Table S1** The distribution of eGDR, waist circumstance, glycosylated haemoglobin A1c, systolic blood pressure and diastolic blood pressure at baseline of participants by sex and median (IQR).
**Table S2** The association between parameters of estimated glucose disposal rate and frailty transitions in the Markov model and HR (95% CI).
**Table S3** The association between parameters of estimated glucose disposal rate and frailty risk or frailty reversibility in the Cox model and HR (95% CI).
**Table S4** Estimated glucose disposal rate with transitions among non‐frailty, frailty and death in the multi‐state Markov model and HR (95% CI).
**Table S5** Associations of estimated glucose disposal rate with frailty risk or its reversibility by Cox regression analysis and HR (95% CI).
**Table S6** Estimated glucose disposal rate with transitions among non‐frailty, frailty and death in the multi‐state Markov model and HR (95% CI).
**Table S7** Associations of estimated glucose disposal rate with frailty risk or its reversibility by Cox regression model and HR (95% CI).

## Data Availability

Data are available to researchers on request for purposes of reproducing the results or replicating the procedure by directly contacting the corresponding author.
