# Supplementary material for: Estimated Glucose Disposal Rate Associated With Risk of Frailty and Likelihood of Reversion
Source: J Cachexia Sarcopenia Muscle. 2025 Apr 17;16(2):e13814. doi: 10.1002/jcsm.13814 (PMC12005398; doi:10.1002/jcsm.13814)
Supplement: Supplementary file 1 — Data S1 Supporting Information. [file JCSM-16-e13814-s002.docx]

| Supplemental Table 1. The 30 items used to construct the frailty index | | | |
| --- | --- | --- | --- |
| No | Description of the item | | Cut-off value |
|  | CHARLS | HRS |  |
| 1 | Self-reported physician diagnosed diabetes | | Yes = 1, No = 0 |
| 2 | Self-reported physician diagnosed heart disease | | Yes = 1, No = 0 |
| 3 | Self-reported physician diagnosed stroke | | Yes = 1, No = 0 |
| 4 | Self-reported physician diagnosed cancer | | Yes = 1, No = 0 |
| 5 | Self-reported physician diagnosed arthritis | | Yes = 1, No = 0 |
| 6 | Self-reported physician diagnosed chronic lung disease | | Yes = 1, No = 0 |
| 7 | Self-reported physician diagnosed any emotional, nervous, or psychiatric problems | | Yes = 1, No = 0 |
| 8 | Self-reported physician diagnosed memory-related disease | | Yes = 1, No = 0 |
| 9 | Self-reported distant eyesight | | Poor or fair = 1, excellent, very good, or good = 0 |
| 10 | Self-reported near eyesight | | Poor or fair = 1, excellent, very good, or good = 0 |
| 11 | Self-reported hearing (while using hearing aid  if appropriate) | | Poor or fair = 1, excellent, very good, or good = 0 |
| 12 | Self-reported general health status | | Poor or fair = 1,  Excellent, very good, or good = 0 |
| 13 | Difficulty with dressing | | Yes = 1, No = 0 |
| 14 | Difficulty with bathing or showering | | Yes = 1, No = 0 |
| 15 | Difficulty with eating | | Yes = 1, No = 0 |
| 16 | Difficulty with getting in and out of bed | | Yes = 1, No = 0 |
| 17 | Difficulty with using the toilet | | Yes = 1, No = 0 |
| 18 | Difficulty with managing money | | Yes = 1, No = 0 |
| 19 | Difficulty with taking medication | | Yes = 1, No = 0 |
| 20 | Difficulty with shopping for groceries | | Yes = 1, No = 0 |
| 21 | Difficulty with preparing meals | | Yes = 1, No = 0 |
| 22 | Mobility: difficulty with walking 100 yards or one block | | Yes = 1, No = 0 |
| 23 | Mobility: difficulty with getting up from a chair after sitting for long periods | | Yes = 1, No = 0 |
| 24 | Mobility: difficulty with climbing several flights of stairs without resting | | Yes = 1, No = 0 |
| 25 | Mobility: difficulty with lifting or carrying weights over 10 pounds/jins | | Yes = 1, No = 0 |
| 26 | Mobility: difficulty with picking up a coin from the table | | Yes = 1, No = 0 |
| 27 | Mobility: difficulty with stooping, kneeling, or crouching | | Yes = 1, No = 0 |
| 28 | Mobility: difficulty with reaching arms above shoulder level | | Yes = 1, No = 0 |
| 29 | Depression: CESD-10 questionnaire | Depression: CESD-8 questionnaire | CESD-10 >10 = 1, ≤10 = 0 in the CHARLS; CESD-8 ≥ 4 = 1, <4 = 0 in the HRS |
| 30 | Cognition: (orientation test score + memory test score + calculation score) / 19 | | Continuous variable, ranging  from 0 to 1 |
| Memory-related disease included Alzheimer’s disease, dementia, organic brain senility, and other serious memory impairment.  Depression was assessed by the Center for Epidemiologic Studies Depression Scale (CESD). In the CHARLS, CESD-10 was used, and the total score ranged from 0 to 30. In the HRS, CESD-8 was used, and the total score ranged from 0 to 8. A higher score indicated more severe depressive symptoms.   The memory score was the average of words which were not recalled in the immediate and delayed word recall tasks. The memory score ranged from 0 to 10. The orientation test comprised 4 questions about the day of the week, the month, the date of the month, and the year. One point was given for each wrong answer, and the range was from 0 to 4. | | | |
| CHARLS, China Health and Retirement Longitudinal Study; HRS, Health and  Retirement Study | | | |
|  | | | |
|  | | | |
|  | | | |
