# Supplementary material for: Estimated Glucose Disposal Rate Associated With Risk of Frailty and Likelihood of Reversion
Source: J Cachexia Sarcopenia Muscle. 2025 Apr 17;16(2):e13814. doi: 10.1002/jcsm.13814 (PMC12005398; doi:10.1002/jcsm.13814)
Supplement: Supplementary file 2 — Figure S1 Selection flow of the study population in the CHARLS. Figure S2 Selection flow of the study population in the HRS. Figure S3 Subgroup analysis of the association between estimated glucose disposal rate and the risk of frailty in Chinese and American populations. Figure S4 Subgroup analysis of the association between estimated glucose disposal rate and the likelihood of frailty reversion in Chinese and American populations. [file JCSM-16-e13814-s001.docx]

**Supplemental Figures**


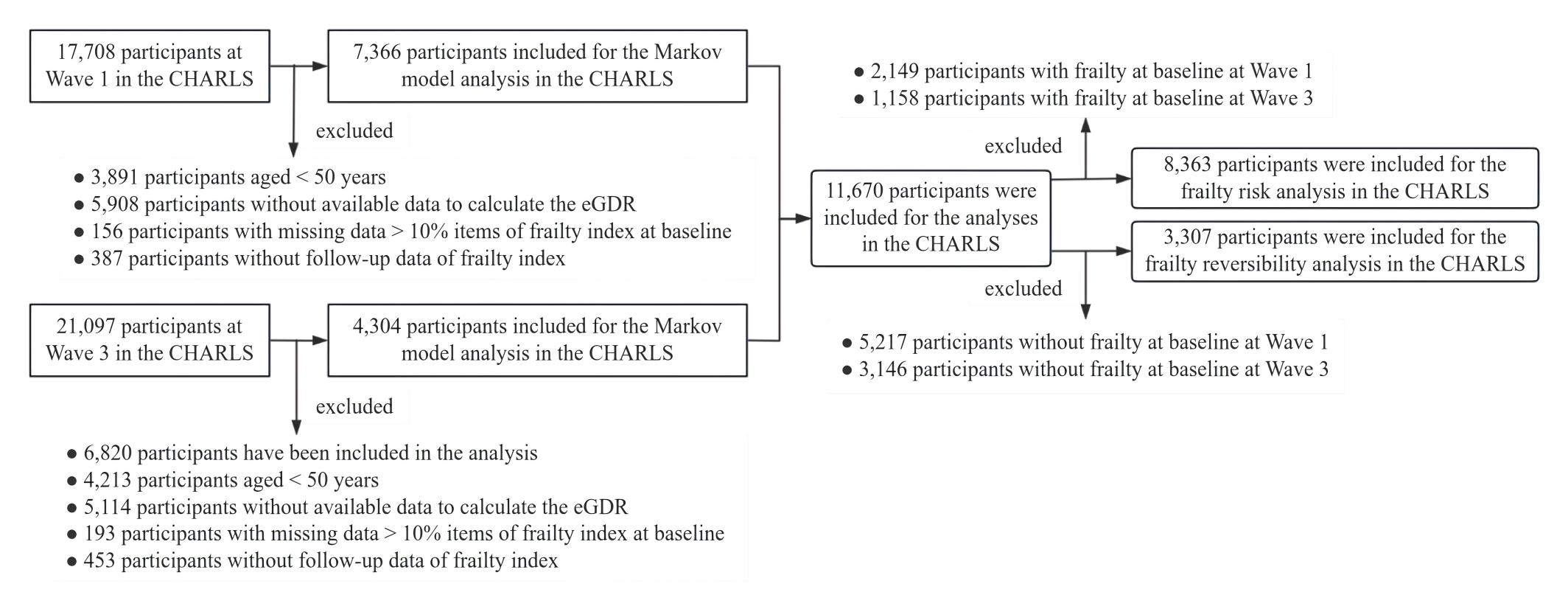


**Figure S1** Selection flow of the study population in the CHARLS.

Notes: CHARLS: China Health and Retirement Longitudinal Study; eGDR: estimated glucose disposal rate


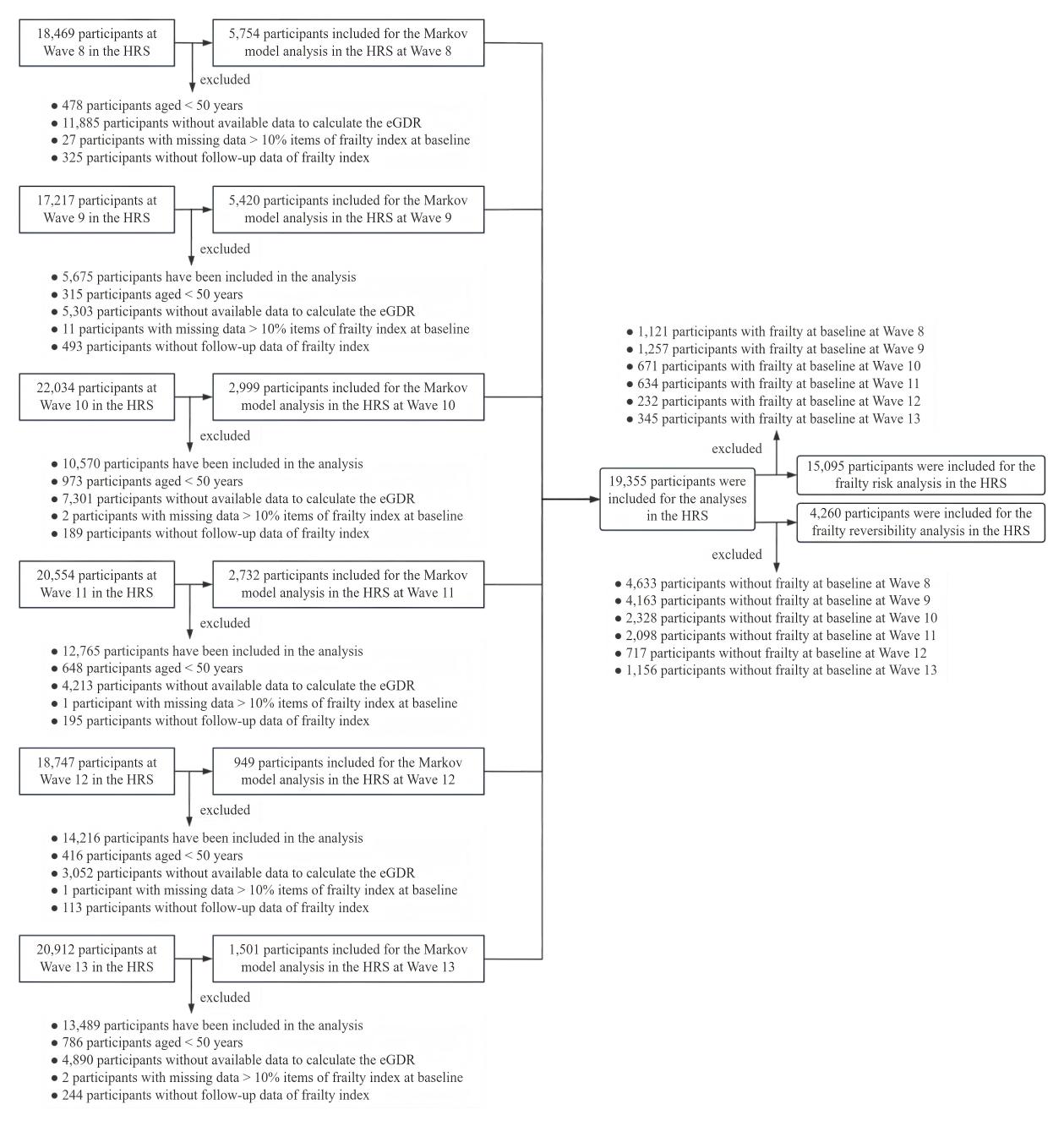


**Figure S2** Selection flow of the study population in the HRS.

Notes: HRS: Health and Retirement Study; eGDR: estimated glucose disposal rate


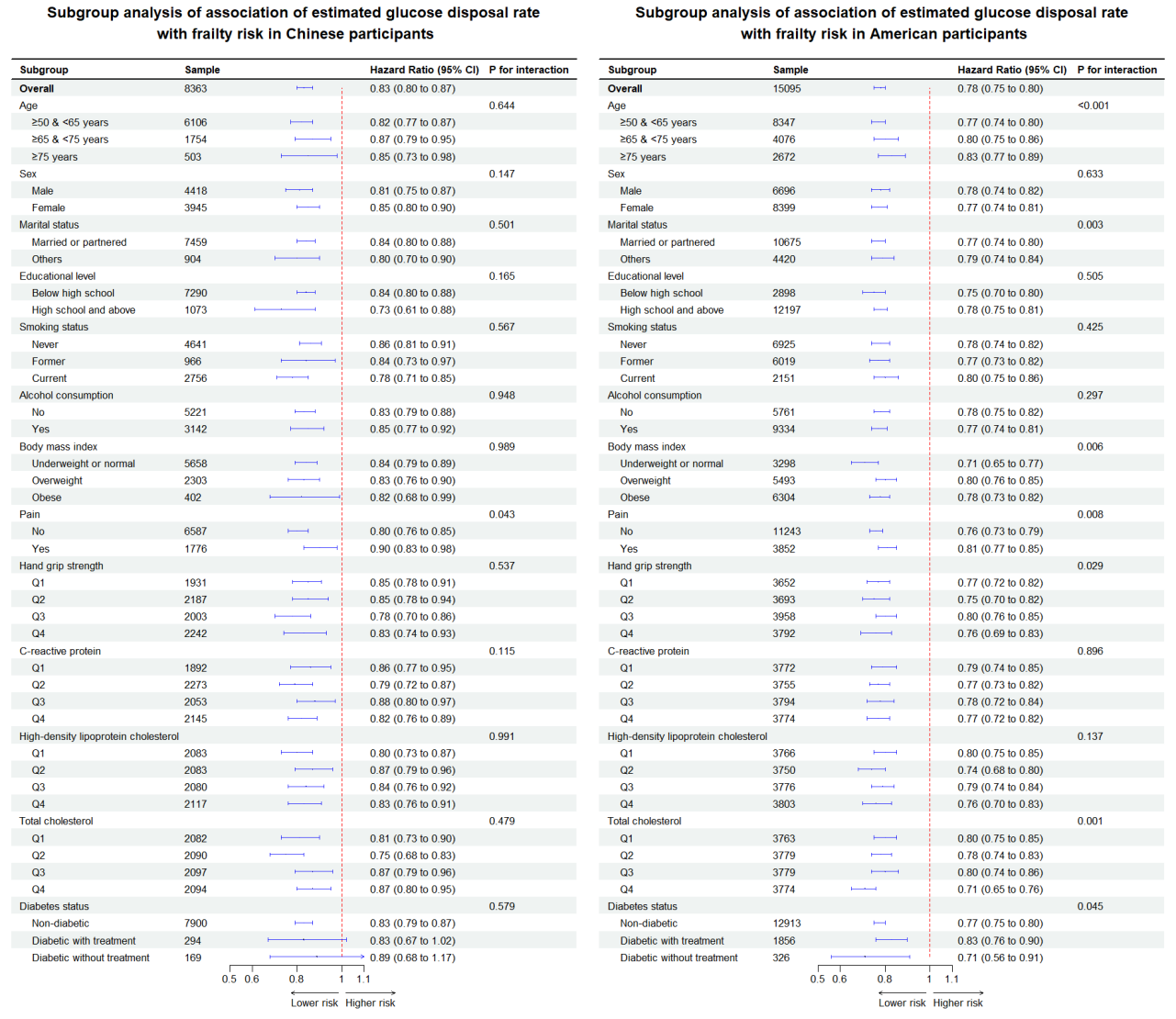


**Figure S3** Subgroup analysis of the association between estimated glucose disposal rate and the risk of frailty in Chinese and American populations.


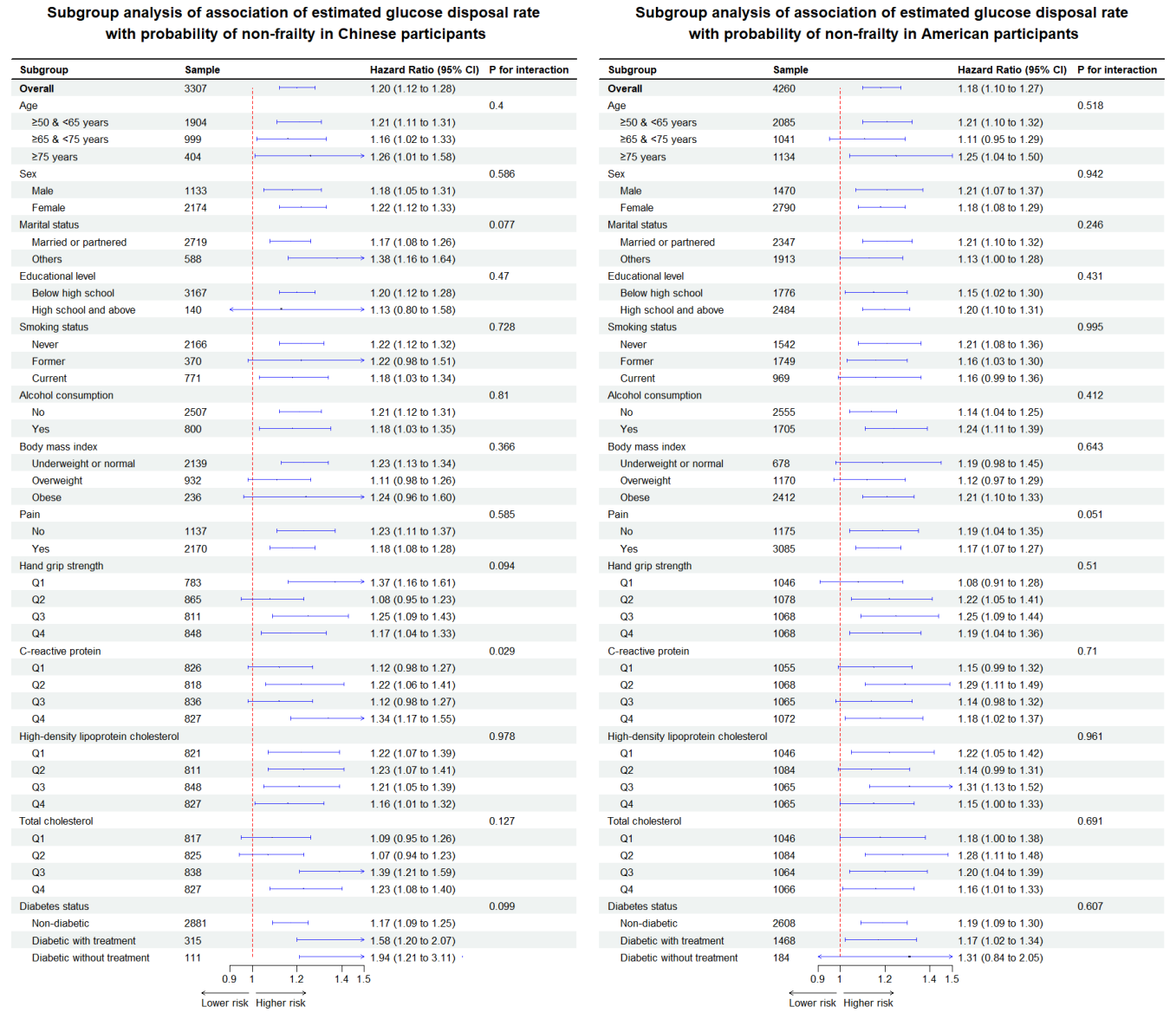


**Figure S4** Subgroup analysis of the association between estimated glucose disposal rate and the likelihood of frailty reversion in Chinese and American populations.
