# Supplementary material for: Estimated Glucose Disposal Rate Associated With Risk of Frailty and Likelihood of Reversion
Source: J Cachexia Sarcopenia Muscle. 2025 Apr 17;16(2):e13814. doi: 10.1002/jcsm.13814 (PMC12005398; doi:10.1002/jcsm.13814)
Supplement: Supplementary file 3 — Table S1 The distribution of eGDR, waist circumstance, glycosylated haemoglobin A1c, systolic blood pressure and diastolic blood pressure at baseline of participants by sex and median (IQR). Table S2 The association between parameters of estimated glucose disposal rate and frailty transitions in the Markov model and HR (95% CI). Table S3 The association between parameters of estimated glucose disposal rate and frailty risk or frailty reversibility in the Cox model and HR (95% CI). Table S4 Estimated glucose disposal rate with transitions among non‐frailty, frailty and death in the multi‐state Markov model and HR (95% CI). Table S5 Associations of estimated glucose disposal rate with frailty risk or its reversibility by Cox regression analysis and HR (95% CI). Table S6 Estimated glucose disposal rate with transitions among non‐frailty, frailty and death in the multi‐state Markov model and HR (95% CI). Table S7 Associations of estimated glucose disposal rate with frailty risk or its reversibility by Cox regression model and HR (95% CI). [file JCSM-16-e13814-s003.docx]

**Table S1** The distribution of eGDR, waist circumstance, glycosylated haemoglobin A1c, systolic blood pressure, and diastolic blood pressure at baseline of participants by sex, median (IQR)

| Characteristics | Chinese participants  (n = 11670) | | | American participants  (n = 19355) | | |
| --- | --- | --- | --- | --- | --- | --- |
|  | Male (n = 5551) | Female (n = 6119) | P value | Male (n = 8166) | Female (n = 11189) | P value |
| Estimated glucose disposal rate (mg/kg/min) | 10.20 (7.77, 11.12) | 9.88 (7.31, 10.90) | < 0.001 | 6.36 (4.70, 8.87) | 6.95 (5.12, 9.54) | < 0.001 |
| Waist circumstance (cm) | 84.50 (78.00, 92.20) | 86.60 (79.20, 93.20) | < 0.001 | 104.14 (96.52, 113.03) | 97.79 (87.63, 109.22) | < 0.001 |
| Glycosylated haemoglobin A1c (%) | 5.30 (5.00, 5.80) | 5.40 (5.10, 5.90) | < 0.001 | 5.68 (5.30, 6.15) | 5.68 (5.30, 6.10) | 0.96 |
| Systolic blood pressure (mmHg) | 127.50 (115.00, 141.50) | 126.50 (113.00, 142.50) | 0.04 | 131.00 (120.00, 144.00) | 125.50 (113.50, 139.50) | < 0.001 |
| Diastolic blood pressure (mmHg) | 75.50 (67.50, 83.50) | 74.00 (66.50, 82.00) | < 0.001 | 80.00 (73.00, 87.50) | 79.00 (72.00, 87.00) | < 0.001 |

Notes: P values obtained through the Wilcoxon rank-sum test.

**Table S2** The association between parameters of estimated glucose disposal rate and frailty transitions in the Markov model, HR (95%CI)

| Transitions | Chinese participants (n = 11670) | | | |  | American participants (n = 19355) | | | |
| --- | --- | --- | --- | --- | --- | --- | --- | --- | --- |
|  | Waist circumference | HbA1c | Systolic blood pressure | Diastolic blood pressure |  | Waist circumference | HbA1c | Systolic blood pressure | Diastolic blood pressure |
| State 1 to state 2 | 1.02 (0.92~1.14) | 0.93 (0.79~1.11) | **1.16 (1.05~1.28)** | 1.00 (0.87~1.15) |  | **1.34 (1.24~1.44)** | **1.16 (1.07~1.26)** | **1.15 (1.08~1.23)** | 1.01 (0.93~1.09) |
| State 1 to state 3 | 0.93 (0.48~1.81) | 0.68 (0.15~3.08) | **1.95 (1.17~3.26)** | 0.66 (0.29~1.53) |  | 1.08 (0.80~1.47) | 0.88 (0.57~1.37) | 1.07 (0.80~1.44) | 1.30 (0.94~1.79) |
| State 2 to state 1 | 0.88 (0.77~1.02) | **0.68 (0.53~0.87)** | 1.03 (0.90~1.17) | 0.83 (0.69~1.00) |  | 0.94 (0.84~1.05) | 1.04 (0.93~1.16) | 0.99 (0.91~1.09) | 0.98 (0.89~1.09) |
| State 2 to state 3 | 0.90 (0.64~1.27) | 1.34 (0.89~2.03) | 1.15 (0.88~1.52) | **1.77 (1.27~2.47)** |  | 0.96 (0.86~1.07) | 1.00 (0.90~1.12) | **1.19 (1.09~1.29)** | 0.95 (0.85~1.06) |

Notes: HR: Hazard ratio; CI: Confidence interval; HbA1c: Glycosylated haemoglobin A1c.

State 1: Non-frailty; State 2: Frailty; State 3: Death.

The waist circumference, HbA1c, systolic blood pressure, and diastolic blood pressure were included in the Markov model as dichotomous variables. The cutoff values are as following: waist circumference (Chinese participants: ≥90 cm in male, ≥85 cm in female; American participants: ≥102 cm in male, ≥88 cm in female), HbA1c (≥6.5%), systolic blood pressure (≥140 mmHg), diastolic blood pressure (≥90 mmHg).

**Table S3** The association between parameters of estimated glucose disposal rate and frailty risk or frailty reversibility in the Cox model, HR (95%CI)

| Parameters | Chinese participants | |  | American participants | |
| --- | --- | --- | --- | --- | --- |
|  | Frailty | Frailty reversibility |  | Frailty | Frailty reversibility |
|  | (n = 8363) | (n = 3307) |  | (n = 15095) | (n = 4260) |
| Waist circumference (cm, per SD) | 1.02 (0.97~1.08) | 0.97 (0.89~1.05) |  | **1.18 (1.15~1.21)** | **0.83 (0.77~0.90)** |
| Normal | Ref. | Ref. |  | Ref. | Ref. |
| Abdominal obesity ^a^ | 1.05 (0.94~1.17) | 0.94 (0.81~1.09) |  | **1.40 (1.29~1.52)** | 0.86 (0.72~1.02) |
| HbA1c (%, per SD) | **1.08 (1.04~1.13)** | **0.80 (0.74~0.87)** |  | **1.11 (1.07~1.14)** | 1.00 (0.94~1.06) |
| <6.5 | Ref. | Ref. |  | Ref. | Ref. |
| ≥6.5 | 1.11 (0.94~1.32) | **0.76 (0.58~0.99)** |  | **1.20 (1.09~1.32)** | 0.98 (0.83~1.15) |
| Systolic blood pressure (mmHg, per SD) | **1.08 (1.02~1.14)** | 0.98 (0.90~1.06) |  | **1.10 (1.05~1.15)** | 0.95 (0.87~1.03) |
| <140 | Ref. | Ref. |  | Ref. | Ref. |
| ≥140 | **1.12 (1.02~1.24)** | 1.01 (0.88~1.16) |  | **1.13 (1.05~1.22)** | 1.00 (0.87~1.15) |
| Diastolic blood pressure (mmHg, per SD) | 1.00 (0.94~1.06) | 0.98 (0.91~1.07) |  | 0.96 (0.92~1.01) | 1.04 (0.96~1.14) |
| <90 | Ref. | Ref. |  | Ref. | Ref. |
| ≥90 | 1.05 (0.91~1.20) | **0.80 (0.65~0.98)** |  | 1.06 (0.97~1.15) | 1.05 (0.89~1.23) |

Notes: HR: Hazard ratio; CI: Confidence interval; SD: Standard deviation; HbA1c: Glycosylated haemoglobin A1c.

^a^ There are sex and race differences in the cutoff values of abdominal obesity (Chinese participants: ≥90 cm in male, ≥85 cm in female; American participants: ≥102 cm in male, ≥88 cm in female).

**Table S4** Estimated glucose disposal rate with transitions among non-frailty, frailty, and death in the multi-state Markov model, HR (95%CI)

| Transitions | Chinese participants (n = 11424) | | | |  | American participants (n = 19241) | | | |
| --- | --- | --- | --- | --- | --- | --- | --- | --- | --- |
|  | Estimated glucose disposal rate | | | |  | Estimated glucose disposal rate | | | |
|  | Q_1_ | Q_2_ | Q_3_ | Q_4_ |  | Q_1_ | Q_2_ | Q_3_ | Q_4_ |
| State 1 to state 2 | Ref. | 1.00 (0.89~1.12) | **0.80 (0.70~0.90)** | **0.83 (0.73~0.94)** |  | Ref. | **0.88 (0.81~0.94)** | **0.79 (0.73~0.85)** | **0.57 (0.52~0.62)** |
| State 1 to state 3 | Ref. | 0.68 (0.31~1.51) | 0.81 (0.39~1.68) | 1.14 (0.55~2.38) |  | Ref. | 0.91 (0.65~1.28) | 0.80 (0.56~1.13) | **0.58 (0.38~0.91)** |
| State 2 to state 1 | Ref. | **1.30 (1.12~1.51)** | **1.41 (1.21~1.66)** | **1.48 (1.25~1.75)** |  | Ref. | **1.33 (1.20~1.46)** | **1.41 (1.27~1.56)** | **1.55 (1.36~1.76)** |
| State 2 to state 3 | Ref. | 0.98 (0.71~1.35) | 0.91 (0.63~1.32) | 1.18 (0.81~1.70) |  | Ref. | 0.91 (0.82~1.01) | 0.99 (0.89~1.11) | 0.95 (0.82~1.10) |

Notes: Multi-state Markov model: using the new frailty index after removing self-reported hypertension and diabetes from the original frailty index; HR: hazard ratio after adjusting for age, sex, marital status, educational level, smoking status, alcohol consumption, body mass index, hand grip strength, pain, C-reactive protein, high-density lipoprotein cholesterol, total cholesterol, diabetes status; CI: confidence interval

State 1: non-frailty; State 2: frailty; State 3: death

**Table S5** Associations of estimated glucose disposal rate with frailty risk or its reversibility by Cox regression analysis, HR (95%CI)

| Estimated glucose  disposal rate | Chinese participants | |  | American participants | |
| --- | --- | --- | --- | --- | --- |
|  | Frailty | Frailty reversibility |  | Frailty | Frailty reversibility |
|  | (n = 8005) | (n = 3419) |  | (n = 14840) | (n = 4401) |
| Q_1_ | Ref. | Ref. |  | Ref. | Ref. |
| Q_2_ | **0.87 (0.78~0.98)** | 1.19 (0.99~1.42) |  | **0.85 (0.78~0.92)** | **1.29 (1.10~1.51)** |
| Q_3_ | **0.77 (0.68~0.87)** | **1.41 (1.18~1.69)** |  | **0.79 (0.72~0.85)** | **1.36 (1.14~1.63)** |
| Q_4_ | **0.74 (0.66~0.84)** | **1.50 (1.23~1.82)** |  | **0.56 (0.51~0.63)** | **1.46 (1.21~1.75)** |

Notes: Cox regression analysis: after removing self-reported hypertension and diabetes from the original frailty index; HR: hazard ratio after adjusting for age, sex, marital status, educational level, smoking status, alcohol consumption, body mass index, hand grip strength, pain, C-reactive protein, high-density lipoprotein cholesterol, total cholesterol, diabetes status; CI: confidence interval

**Table S6** Estimated glucose disposal rate with transitions among non-frailty, frailty, and death in the multi-state Markov model, HR (95%CI)

| Transitions | Chinese participants (n = 10760) | | | |  | American participants (n = 15461) | | | |
| --- | --- | --- | --- | --- | --- | --- | --- | --- | --- |
|  | Estimated glucose disposal rate | | | |  | Estimated glucose disposal rate | | | |
|  | Q_1_ | Q_2_ | Q_3_ | Q_4_ |  | Q_1_ | Q_2_ | Q_3_ | Q_4_ |
| State 1 to state 2 | Ref. | 0.93 (0.83~1.04) | **0.78 (0.68~0.88)** | **0.74 (0.65~0.85)** |  | Ref. | **0.78 (0.72~0.84)** | **0.75 (0.70~0.82)** | **0.52 (0.47~0.58)** |
| State 1 to state 3 | Ref. | 0.63 (0.27~1.48) | 0.83 (0.39~1.75) | 1.47 (0.74~2.92) |  | Ref. | 0.98 (0.67~1.45) | 0.77 (0.52~1.13) | **0.58 (0.36~0.93)** |
| State 2 to state 1 | Ref. | **1.29 (1.10~1.50)** | **1.27 (1.08~1.49)** | **1.25 (1.06~1.48)** |  | Ref. | 1.12 (1.00~1.26) | **1.33 (1.19~1.50)** | **1.39 (1.20~1.61)** |
| State 2 to state 3 | Ref. | 0.88 (0.62~1.23) | 0.89 (0.62~1.29) | 0.92 (0.64~1.34) |  | Ref. | 0.95 (0.84~1.07) | 1.03 (0.91~1.17) | 0.98 (0.83~1.16) |

Notes: Multi-state Markov model: after excluding participants suffering from diabetes at baseline; HR: hazard ratio after adjusting for age, sex, marital status, educational level, smoking status, alcohol consumption, body mass index, hand grip strength, pain, C-reactive protein, high-density lipoprotein cholesterol, total cholesterol; CI: confidence interval

State 1: non-frailty; State 2: frailty; State 3: death

**Table S7** Associations of estimated glucose disposal rate with frailty risk or its reversibility by Cox regression model, HR (95%CI)

| Estimated glucose  disposal rate | Chinese participants | |  | American participants | |
| --- | --- | --- | --- | --- | --- |
|  | Frailty | Frailty reversibility |  | Frailty | Frailty reversibility |
|  | (n = 7880) | (n = 2880) |  | (n = 12871) | (n = 2590) |
| Q_1_ | Ref. | Ref. |  | Ref. | Ref. |
| Q_2_ | **0.80 (0.72~0.90)** | 1.13 (0.94~1.36) |  | **0.83 (0.76~0.91)** | **1.30 (1.06~1.59)** |
| Q_3_ | **0.78 (0.69~0.88)** | **1.35 (1.12~1.62)** |  | **0.74 (0.68~0.81)** | **1.46 (1.18~1.80)** |
| Q_4_ | **0.72 (0.63~0.81)** | **1.40 (1.15~1.71)** |  | **0.56 (0.50~0.63)** | **1.56 (1.25~1.94)** |

Notes: Cox regression analysis: after excluding participants suffering from diabetes at baseline; HR：hazard ratio after adjusting for age, sex, marital status, educational level, smoking status, alcohol consumption, body mass index, hand grip strength, pain, C-reactive protein, high-density lipoprotein cholesterol, total cholesterol; CI: confidence interval
